# Blood Metabolomic Profiling of Systemic Responses to Dried Black Lychee in a Scopolamine-Induced Cognitive Impairment in Rats

**DOI:** 10.3390/biology15151325

**Published:** 2026-08-06

**Authors:** Punate Weerateerangkul, Napapan Kangwan, Watcharaporn Preedapirom Jeefoo, Anongporn Kobroob, Giatgong Konguthaithip, Kanicnan Intui, Somlada Watcharakhom, Kanokwan Kulprachakarn, Supakit Chaipoot, Wason Parklak, Hataichanok Chuljerm, Supitchar Samoechai, Nutta Piengjaikum, Sirikorn Namdech, Jiraporn Laoung-on, Churdsak Jaikang

**Affiliations:** 1 Department of Physiology, Faculty of Medicine, Chiang Mai University, Chiang Mai 50200, Thailand; punate.w@cmu.ac.th; 2 Division of Physiology, School of Medical Science, University of Phayao, Phayao 56000, Thailand; napapan.ka@up.ac.th (N.K.); watcharaporn.pr@up.ac.th (W.P.J.); anongporn.ko@up.ac.th (A.K.); 3 Metabolomic Research Group for Forensic Medicine and Toxicology, Department of Forensic Medicine, Faculty of Medicine, Chiang Mai University, Chiang Mai 50200, Thailand; kongkiat.shang@gmail.com (G.K.); supitchar.samoechai@gmail.com (S.S.); nuttapjk2003@gmail.com (N.P.); sirikornea@gmail.com (S.N.); 4 Department of Forensic Medicine, Faculty of Medicine, Chiang Mai University, Chiang Mai 50200, Thailand; kanicnan.i@cmu.ac.th (K.I.); somlada.wat@cmu.ac.th (S.W.); 5 School of Health Sciences Research, Research Institute for Health Sciences, Chiang Mai University, Chiang Mai 50200, Thailand; kanokwan.kul@cmu.ac.th (K.K.); wason.p@cmu.ac.th (W.P.); hataichanok.ch@cmu.ac.th (H.C.); 6 Multidisciplinary Research Institute, Chiang Mai University, Chiang Mai 50200, Thailand; supakit.ch@cmu.ac.th; 7 Research Institute for Health Sciences (RIHES), Chiang Mai University, Chiang Mai 50200, Thailand; jiraporn.l@cmu.ac.th

**Keywords:** black lychee, cognitive impairment, scopolamine, metabolomics

## Abstract

Cognitive impairment and memory decline represent major health concerns affecting older adults worldwide, and systemic metabolic dysfunction plays a critical role in their progression. Black lychee is produced from *Litchi chinensis* Sonn. through postharvest thermal processing. It contains polyphenolic compounds known for their neuroprotective properties. This study investigated the cognitive-enhancing effects of black lychee in a rat model of scopolamine-induced cognitive impairment. Male rats (*n* = 8 per group) received scopolamine (2 mg/kg, i.p.) alone or in combination with black lychee at 100, 200, or 400 mg/kg/day for 20 days. Untargeted blood metabolites and the phytochemical composition of black lychee were identified using ^1^H-NMR spectroscopy. Cognitive performance was assessed using the passive avoidance test. Phytochemical analysis confirmed that black lychee is rich in polyphenols. All black lychee-treated groups showed a significant increase in step-through latency compared with the scopolamine group. Blood metabolomic analysis revealed alterations in 26 metabolic pathways associated with oxidative stress and energy metabolism. These findings suggest that polyphenols in black lychee attenuate scopolamine-induced cognitive impairment by reducing oxidative stress and restoring key metabolites involved in energy metabolism. These results support the potential of black lychee as a functional food for mitigating age-related cognitive decline.

## Simple Summary

Memory loss is a major health problem and affects older people worldwide. Various treatments can relieve symptoms, but they cannot stop disease progression. Therefore, novel preventive and therapeutic approaches are urgently needed. In this study, we investigated black lychee, a new functional food, for its protective effects against memory impairment in scopolamine-treated rats. Rats that received black lychee showed significant improvements in learning and memory. Blood metabolites related to oxidative stress, kynurenine, and energy metabolism were associated with improved cognitive performance. These findings demonstrate the neuroprotective potential of black lychee through the modulation of biological pathways. This emphasizes its potential role as a functional food for maintaining brain health and guiding future strategies to prevent age-related memory decline.

## 1. Introduction

Cognitive impairment is characterized by deficits in learning, memory, attention, and executive function and is commonly observed in older adults [1]. It has been associated with several metabolic abnormalities, including oxidative stress [2], mitochondrial dysfunction [3], impaired energy metabolism [4], dysregulated amino acid metabolism, and altered lipid metabolism [5,6]. These metabolic changes can lead to neuronal dysfunction, synaptic damage, and cognitive decline [5]. Therefore, understanding these metabolic changes may help identify potential targets for preventing or alleviating cognitive impairment.

Although cognitive impairment primarily affects the central nervous system, direct analysis of brain tissue is not feasible in most experimental and clinical settings [7]. Peripheral blood has emerged as a practical and minimally invasive biospecimen for investigating systemic metabolic alterations associated with cognitive impairment [8]. Blood metabolomics provides a valuable approach for identifying metabolic signatures, investigating metabolic alterations, and evaluating therapeutic responses [9]. Among the available analytical platforms, untargeted ^1^H-NMR metabolomics enables comprehensive profiling of circulating metabolites and simultaneous assessment of multiple metabolic pathways, including oxidative stress, amino acid, lipid, and energy metabolism [10].

*Litchi chinensis* Sonn., a tropical fruit valued for its high nutritional quality, is rich in bioactive phytochemicals, including polyphenols, flavonoids, phenolic acids, anthocyanins, and proanthocyanidins [11,12]. These compounds exhibit a broad spectrum of biological activities, including antioxidant, anti-inflammatory, anti-apoptotic, anti-amyloidogenic, and acetylcholinesterase inhibitory effects [13,14]. To enhance these health benefits, postharvest thermal processing is applied to produce black lychee as a value-added functional food. The resulting browning reactions alter its phytochemical composition, significantly boosting total phenolic content and antioxidant capacity compared with fresh lychee [15,16]. This enriched phytochemical profile may contribute to the biological activities of black lychee, particularly its antioxidant and neuroprotective properties.

Scopolamine is widely used to induce cognitive impairment in animal models, leading to deficits in learning and memory that mimic key behavioral features of dementia [17]. Beyond cognitive decline, scopolamine administration triggers systemic alterations, including elevated oxidative stress, metabolic dysregulation, and neuroinflammation [18,19]. While previous studies on lychee and its products have mainly evaluated its general antioxidant properties or basic behavioral outcomes, comprehensive insights into the systemic metabolic shifts accompanying its neuroprotective effects remain limited. To address this gap, the present study evaluated the cognitive-enhancing effects of black lychee in a rat model of scopolamine-induced cognitive impairment through behavioral assessment (passive avoidance test) combined with untargeted ^1^H-NMR blood metabolomics. This study aimed to characterize the systemic metabolic responses associated with its cognitive-enhancing effects and to support the development of black lychee as a functional food for mitigating cognitive decline.

## 2. Materials and Methods

### 2.1. Chemical and Plant Material

Scopolamine hydrobromide, N-acetyl-L-cysteine (NAC), 3-(trimethylsilyl) propionic-2,2,3,3-d_4_ acid sodium salt (TSP, NMR grade), and deuterium oxide (D_2_O, 99.9 atom% D) were purchased from Sigma-Aldrich (St. Louis, MO, USA). Black lychee was provided by Dr. Supakit Chaipoot, Multidisciplinary Research Institute, Chiang Mai University, Chiang Mai 50200, Thailand.

### 2.2. Phytochemical Profile of Black Lychee

The phytochemical composition of the black lychee was characterized using a Bruker AVANCE III 500 MHz NMR spectrometer (Bruker BioSpin, Rheinstetten, Germany). Approximately 5 mg of the dried black lychee was dissolved in 600 μL of D_2_O, vortexed, centrifuged at 15,000 rpm for 5 min, and transferred into a 5 mm NMR tube. Spectra were acquired using 0.05% TSP as an internal standard [20]. A total of 64 scans were collected with 32,768 data points, a spectral width of 12 ppm (or 8278 Hz), an acquisition time of 3.95 s, and a relaxation delay of 2.0 s. Free induction decays (FID) were Fourier transformed following exponential line broadening of 0.3 Hz. Spectra were manually phase- and baseline-corrected using TopSpin software (version 4.0.7; Bruker BioSpin, Rheinstetten, Germany). Compounds were identified based on chemical shifts, signal multiplicities, and J-coupling constants by comparison with reference spectra in the Human Metabolome Database (HMDB). All assignments were manually verified and accepted only when chemical shift values agreed within ± 0.01 ppm of the reported reference values [21].

### 2.3. Treatment and Experimental Procedures in Animals

All experimental procedures involving animals were approved by the Ethics Committee of the Laboratory Animal Research Center, University of Phayao, Thailand (Approval No. 1-045-68, approved 26 December 2025) and were conducted in accordance with institutional guidelines for the care and use of laboratory animals. Male BrlHan:Wist@Jcl (GALAS) rats (aged 5–7 weeks, weighing approximately 200 g) were obtained from Nomura Siam International Co., Ltd. (Bangkok, Thailand). All animals underwent a health examination covering general appearance, body weight, and physical condition and were quarantined for at least 7 days to acclimatize and monitor for signs of illness or abnormal behavior.

**Figure 1:**
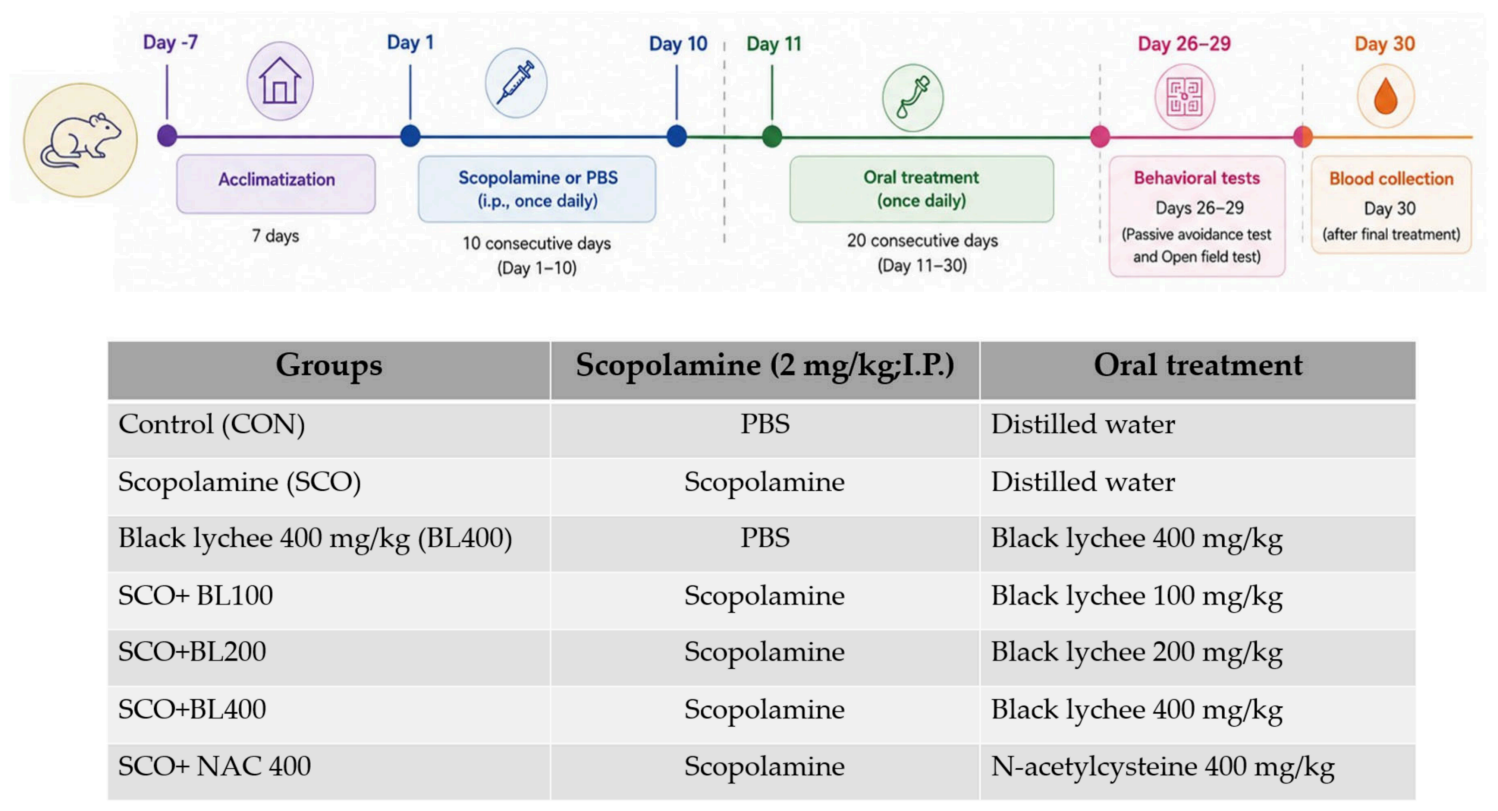
Schematic illustration of the experimental protocol. After a 7-day acclimatization period, rats were randomly allocated into seven groups (n = 8 per group). Scopolamine (2 mg/kg, i.p.) or phosphate-buffered saline pH 7.4 (PBS) was administered for 10 consecutive days, followed by oral administration of black lychee (100–400 mg/kg/day), NAC (400 mg/kg/day), or distilled water for 20 days. Behavioral tests were performed during the last four days of treatment before blood collection on Day 30 for metabolomic analysis.

After acclimatization, the rats were housed under standard laboratory conditions (22 ± 2 °C, 50–60% relative humidity, and a 12 h light/12 h dark cycle) with standard chow and water provided ad libitum. Rats were then randomly assigned to seven experimental groups (n = 8 per group), as illustrated in Figure 1. Humane endpoints were predefined and included a body weight loss exceeding 20%, inability to access food or water, persistent distress or moribund state, severe lethargy, and self-inflicted injury; animals meeting these criteria were euthanized immediately regardless of the study timepoint. At the end of the study, animals were euthanized by an intraperitoneal overdose of sodium pentobarbital (50 mg/kg) in accordance with institutional standard operating procedures.

### 2.4. Open-Field Test and Passive Avoidance Test

To determine whether differences in cognitive performance were influenced by motor impairment, locomotor activity was evaluated using the open-field test. The apparatus consisted of a transparent acrylic box (72 × 72 × 36 cm) with the floor divided into 16 equal squares (18 × 18 cm). Each rat was placed in the center of the arena and allowed to explore freely for 5 min [22]. The number of line crossings, defined as the number of times the rat crossed from one square to another with all four paws, was recorded as an index of locomotor activity. Comparable locomotor activity among groups indicated that subsequent cognitive assessments were not affected by motor deficits. All behavioral experiments were performed between 09:00 and 12:00 h in a quiet room under constant illumination (approximately 100 lux). The apparatus was cleaned with 70% ethanol between animals to eliminate olfactory cues. Animal behavior was recorded using a digital video camera and analyzed by an investigator blinded to treatment allocation.

Learning and memory were assessed using the passive avoidance test. The apparatus consisted of two interconnected acrylic chambers (20 × 20 × 40 cm each) separated by a sliding door: one brightly illuminated chamber (light compartment) and one dark chamber (dark compartment) equipped with a stainless-steel grid floor connected to an electric shock generator. During the acquisition trial, each rat was placed in the illuminated compartment. When the rat entered the dark compartment, the sliding door was closed, and a foot shock (0.5 mA for 3 s) was delivered through the stainless-steel grid floor. Twenty-four hours later, memory retention was evaluated in the absence of foot shock by measuring step-through latency. A maximum latency of 300 s was used as the cutoff time, with longer latency indicating better memory retention. The apparatus was cleaned with 70% ethanol between animals, and all behavioral assessments were performed by an investigator blinded to treatment allocation [23].

### 2.5. Blood Sample Collection

At the end of the experimental period, rats were anesthetized with sodium pentobarbital (20 mg/kg, i.p.). Blood was collected from the abdominal aorta into heparin tubes. Plasma was separated by centrifugation at 3000× *g*, 10 min at 4 °C, aliquoted, snap-frozen, and stored at −80 °C until biochemical and ^1^H-NMR metabolomic analyses.

### 2.6. Blood Sample Extraction for ^1^H-NMR Analysis

Blood samples were deproteinized by mixing with acetonitrile at a 1:1 (v/v) ratio, followed by centrifugation at 4000 rpm for 10 min at 4 °C. The supernatant was collected and lyophilized. The dried residue was reconstituted in 0.6 mL of D_2_O containing 1 µM TSP as a chemical shift reference, transferred into 5 mm NMR tubes, and analyzed using a 500 MHz ^1^H-NMR spectrometer under the acquisition conditions described in Section 2.2 [24].

### 2.7. NMR Data Acquisition

^1^H-NMR spectra were acquired on a Bruker AVANCE III 500 MHz spectrometer (Bruker BioSpin, Rheinstetten, Germany) at 27 °C using the Carr–Purcell–Meiboom–Gill (CPMG) pulse sequence to suppress broad signals from macromolecules. Water suppression was achieved by presaturation during the relaxation delay period. Acquisition parameters were as follows: 16 scans, relaxation delay 1.0 s, acquisition time 3.95 s, spectral width 8278.146 Hz, dwell time 60.40 μs, and 90° pulse angle. FID data were processed in TopSpin (version 4.0.7, Bruker BioSpin, Germany) with manual phase and baseline corrections. All spectra were referenced to TSP (δ 0.00 ppm) as the internal chemical shift standard.

Metabolite annotation was performed based on evaluation of ^1^H-NMR spectral characteristics, including chemical shifts, signal multiplicity, coupling patterns (J-coupling), peak shape, signal integration, and the consistency of resonance assignments within individual metabolites. Assignments were further supported by comparison with reference spectra available in the Human Metabolome Database (HMDB). Biological plausibility and consistency with previously reported metabolic pathways were also considered during metabolite annotation.

### 2.8. Data Processing and Statistical Analysis

Processed spectra were analyzed over the chemical shift range of 0.00–12.00 ppm. Metabolite annotation was performed by comparison with reference data from the HMDB (accessed May 2026) based on chemical shifts, signal multiplicity patterns, and J-coupling constants. All peak assignments were manually verified with a chemical shift difference within ±0.01 ppm of the reported reference values.

Peak integration was performed using MNova software (version 12.0.0, Mestrelab Research, Santiago de Compostela, Spain). The TSP signal at 0.00 ppm was used for spectral calibration and normalization. Relative metabolite levels were calculated according to the following equation:

$$\frac{I_{A}}{I_{B}}=\frac{H_{A}}{H_{B}}\times \frac{C_{A}}{C_{B}}$$

where *I* represents the integrated peak intensity, H represents the number of contributing protons, and C represents the relative metabolite concentration.

Metabolomic data were analyzed using MetaboAnalyst 6.0 (https://www.metaboanalyst.ca) [25]. Missing values were imputed prior to statistical analysis, followed by median normalization, log_10_ transformation, and auto-scaling. Unsupervised principal component analysis (PCA) and supervised partial least squares-discriminant analysis (PLS-DA) were performed to evaluate overall metabolic patterns and group separation. Variable importance in projection (VIP) scores were calculated to identify metabolites contributing to group discrimination. Pathway enrichment analysis was conducted to identify altered metabolic pathways.

### 2.9. Statistical Analysis

Data are presented as mean ± standard deviation (SD). Differences among multiple groups were assessed using the Kruskal–Wallis test, followed by Dunn’s post hoc test for pairwise comparisons. Correlations between retention test scores at 24 h and blood metabolite levels were evaluated using Spearman’s rank correlation. A *p*-value of less than 0.05 (*p* < 0.05) was considered statistically significant.

## 3. Results

### 3.1. Phytochemical Contents in Black Lychee

**Figure 2:**
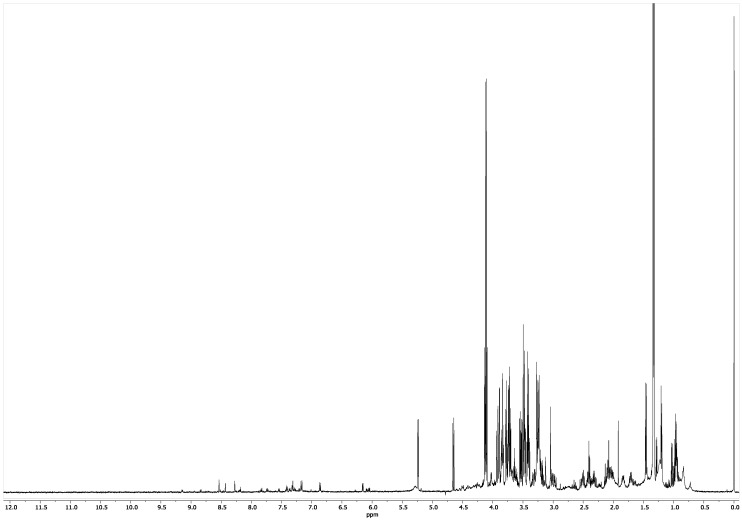
The ^1^H-NMR chromatogram of black lychee. The chemical shift at 1.38 = rutin, 4.56 = catechin, 4.95 = epicatechin, 5.52 = epigallocatechin gallate, 6.71 = isoquercitrin, 6.78 = meloside, 6.84 = flavone, 7.04 = quercetin 4′-glycoside, 7.29 = gallic acid, 7.33 = coumarin acid, 7.53 = cyanidin, 7.55 = caffeic acid, 7.68 = quercetin, 8.08 = kaempferol, 8.58 = delphinidin 3-glucoside, 8.70 = procyanidin B2, and 11.90 = taxifolin.

The phytochemical composition of black lychee was characterized by ^1^H-NMR spectroscopy. The NMR spectrum is demonstrated in Figure 2. A total of seventeen major phytochemicals were identified based on their chemical shifts, signal multiplicities, and comparison with the HMDB. The identified metabolites were predominantly polyphenolic compounds, including flavan-3-ols (catechin, epicatechin, epigallocatechin gallate, and procyanidin B2), flavonols (quercetin, kaempferol, isoquercitrin, rutin, quercetin 4′-glycoside, melitoside, and taxifolin), anthocyanins (cyanidin-3-glucoside and delphinidin-3-glucoside), and phenolic acids (gallic acid, caffeic acid, and p-coumaric acid). Moreover, flavone derivatives were also detected in black lychee.

### 3.2. General Characteristics of Experimental Animals

The absence of significant differences in body weight and general health status among all experimental groups indicates that neither scopolamine nor black lychee treatment induced overt systemic toxicity during the study period. These findings align with previous studies employing scopolamine-induced cognitive impairment models, where memory deficits occurred without marked alterations in body weight or general physical condition. This suggests that the observed behavioral deficits were primarily driven by central cholinergic dysfunction rather than generalized malaise or motor debilitation. Furthermore, the lack of adverse effects following black lychee administration demonstrates its favorable tolerability profile, suggesting that the observed cognitive enhancements were specifically mediated by its bioactive properties rather than confounding shifts in overall animal health.

### 3.3. Effect of Black Lychee on Locomotor Activity and Learning and Memory

Locomotor activity was evaluated using the open-field test by recording total line crossings over a 5 min period. As presented in Table 1, no significant differences were observed among the experimental groups, indicating that neither scopolamine nor black lychee treatment altered spontaneous motor activity. These results rule out motor dysfunction as a confounding factor in the subsequent cognitive evaluations. Learning and memory retention were assessed using the step-through passive avoidance test. During the training session, step-through latencies were comparable across all groups, indicating uniform baseline performance prior to memory acquisition. However, in the 24 h retention test, the scopolamine-treated group exhibited a significantly shorter step-through latency compared with the control group (*p* < 0.05), reflecting marked memory impairment. In contrast, black lychee treatment in all doses significantly prolonged the step-through latency relative to the scopolamine group, suggesting an improvement in learning and memory. Similarly, the NAC-treated group showed a significantly longer latency than the scopolamine group.

### 3.4. Effect of Black Lychee on Blood Metabolic Profiles

**Figure 3:**
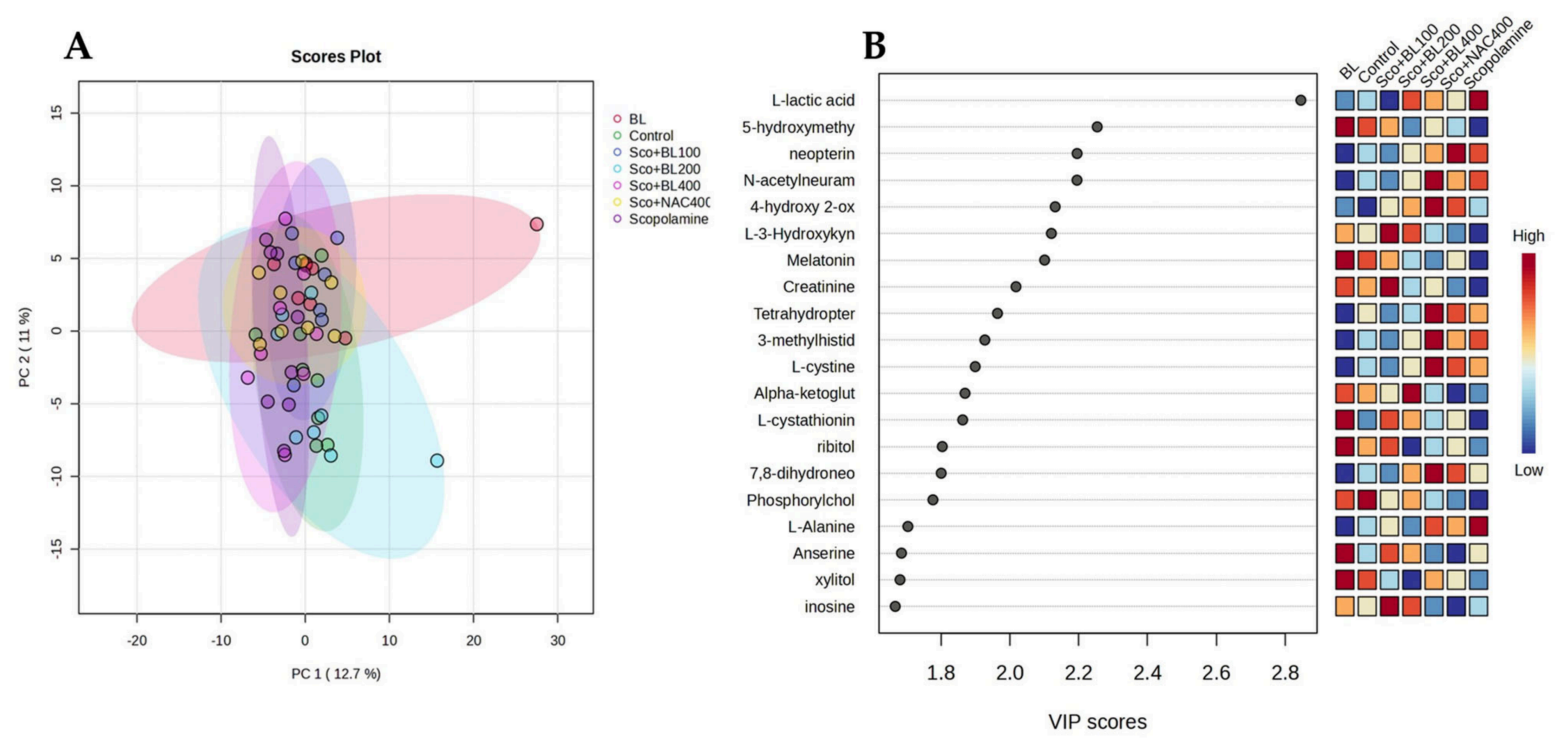
Principal component analysis (PCA) score plot (**A**) and variable importance in projection (VIP) score plot (**B**) of the experimental groups.

Principal component analysis (PCA) was performed to investigate overall profile variations among the seven experimental groups. The PCA score plot (Figure 3A) demonstrates that samples are distributed along the first two principal components, which together account for 23.7% of the total variance (PC1 = 12.7% and PC2 = 11.0%). Permutational multivariate analysis of variance (PERMANOVA; F = 2.67, *p* = 0.001; 999 permutations) was conducted to determine whether differences among treatment groups were statistically significant despite partial visual overlap on the PCA plot. The PERMANOVA results revealed a significant difference in global metabolic profiles across groups. An *R*^2^ value of 0.250 indicates that approximately 25.0% of the total variance was attributable to treatment conditions, with the remaining variation arising from within-group variability. The PLS-DA model was evaluated using internal cross-validation and permutation testing. The model showed an R^2^ value of 0.7149 and a Q^2^ value of 0.3186 (Supplementary Figure S1). Variable importance in projection (VIP) analysis was subsequently performed to identify key discriminative metabolites driving group separation (Figure 3B). A total of 20 metabolites with high VIP scores (VIP > 1.5) were identified. Among these, L-lactic acid emerged as the most influential biomarker, displaying the highest VIP score, followed by 5-hydroxymethylfurfural, neopterin, and N-acetylneuraminate, respectively. Notably, 5-hydroxymethylfurfural, melatonin, and L-cystathionine exhibited elevated relative levels (red) in the control and BL-treated groups but were markedly depleted (blue) in the scopolamine-treated group. L-lactic acid levels were decreased in the SCO + BL100 group, whereas they were elevated in the scopolamine, SCO + BL200, and SCO + BL400 groups. These results demonstrate that the experimental treatments induced statistically significant and metabolite-specific alterations in the overall profile.

**Figure 4:**
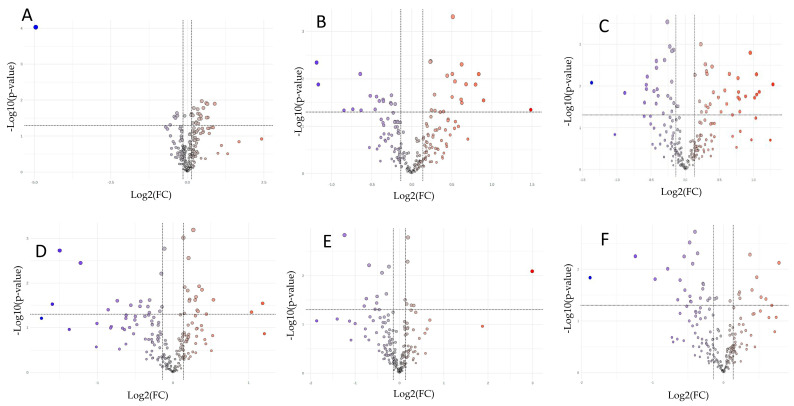
Volcano plot analysis of differential blood metabolites in the experimental groups compared with the control group: (**A**) control vs. scopolamine (SCO); (**B**) control vs. black lychee (BL400); (**C**) control vs. SCO + BL100; (**D**) control vs. SCO + BL200; (**E**) control vs. SCO + BL400; and (**F**) control vs. SCO + N-acetyl-L-cysteine (NAC). Each point represents one metabolite detected by ^1^H-NMR spectroscopy. Red and blue dots indicate significantly increased and decreased metabolites, respectively. The horizontal dashed line represents the significance threshold (*p* < 0.05), and the vertical dashed lines indicate the fold-change threshold.

To investigate the effect of black lychee on metabolites, volcano plot analysis was performed comparing each treatment group with the control group (Figure 4). Scopolamine treatment altered the blood metabolomic profile (Figure 4A). However, administration of black lychee alone (400 mg/kg) resulted in relatively few metabolic changes (Figure 4B). All doses of black lychee supplementation changed the metabolic profile in scopolamine-treated rats (Figure 4C–E). The metabolites involved in oxidative stress, kynurenine, and energy metabolism were altered in the black lychee-treated groups (Supplementary Table S1). Treatment with NAC also attenuated scopolamine-induced metabolic disturbances (Figure 4F).

**Figure 5:**
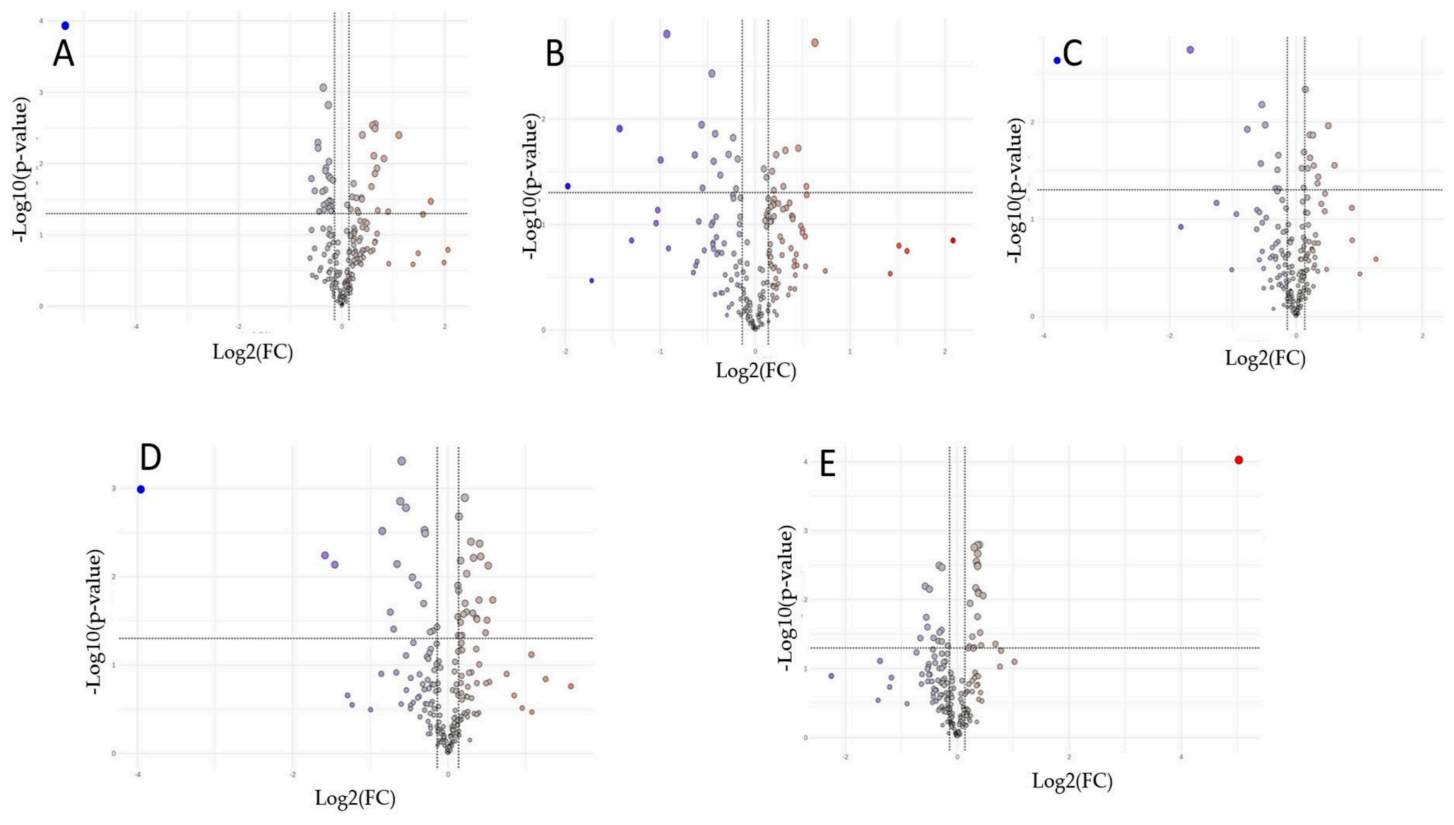
Volcano plot analysis of differential blood metabolites between the scopolamine group and treatment groups: (**A**) SCO vs. BL400; (**B**) SCO vs. SCO + BL100; (**C**) SCO vs. SCO + BL200; (**D**) SCO vs. SCO + BL400; and (**E**) SCO vs. SCO + N-acetyl-L-cysteine (NAC). Each point represents one metabolite detected by ^1^H-NMR spectroscopy. Red and blue dots indicate significantly increased and decreased metabolites, respectively. The horizontal dashed line represents the significance threshold (*p* < 0.05), and the vertical dashed lines indicate the fold-change threshold.

Volcano plot analysis was performed to identify differential blood metabolites between the SCO group and each treatment group (Figure 5). Compared with SCO, treatment with BL400 alone altered relatively few metabolites, with only one metabolite significantly decreased (Figure 5A). In contrast, SCO + BL100 induced the greatest number of significant metabolic alterations, with several metabolites both increased and decreased relative to SCO (Figure 5B). SCO + BL200 and SCO + BL400 similarly showed metabolite-specific changes, each with one markedly decreased metabolite of high significance (Figure 5C,D). Treatment with NAC (SCO + NAC) resulted in one metabolite that was significantly increased relative to SCO, while most other metabolites remained unchanged (Figure 5E). These findings indicate that black lychee supplementation, particularly at lower to moderate doses, and NAC treatment differentially modulated the scopolamine-induced blood metabolomic profile (Supplementary Table S2). Moreover, phytochemicals present in the blood of the treatment groups were analyzed. The identified phenolic compounds included catechin, epicatechin, epigallocatechin gallate, flavone, gallic acid, caffeic acid, quercetin, and kaempferol. The results are presented in Supplementary Table S3.

### 3.5. Pathway Impact Analysis

**Figure 6:**
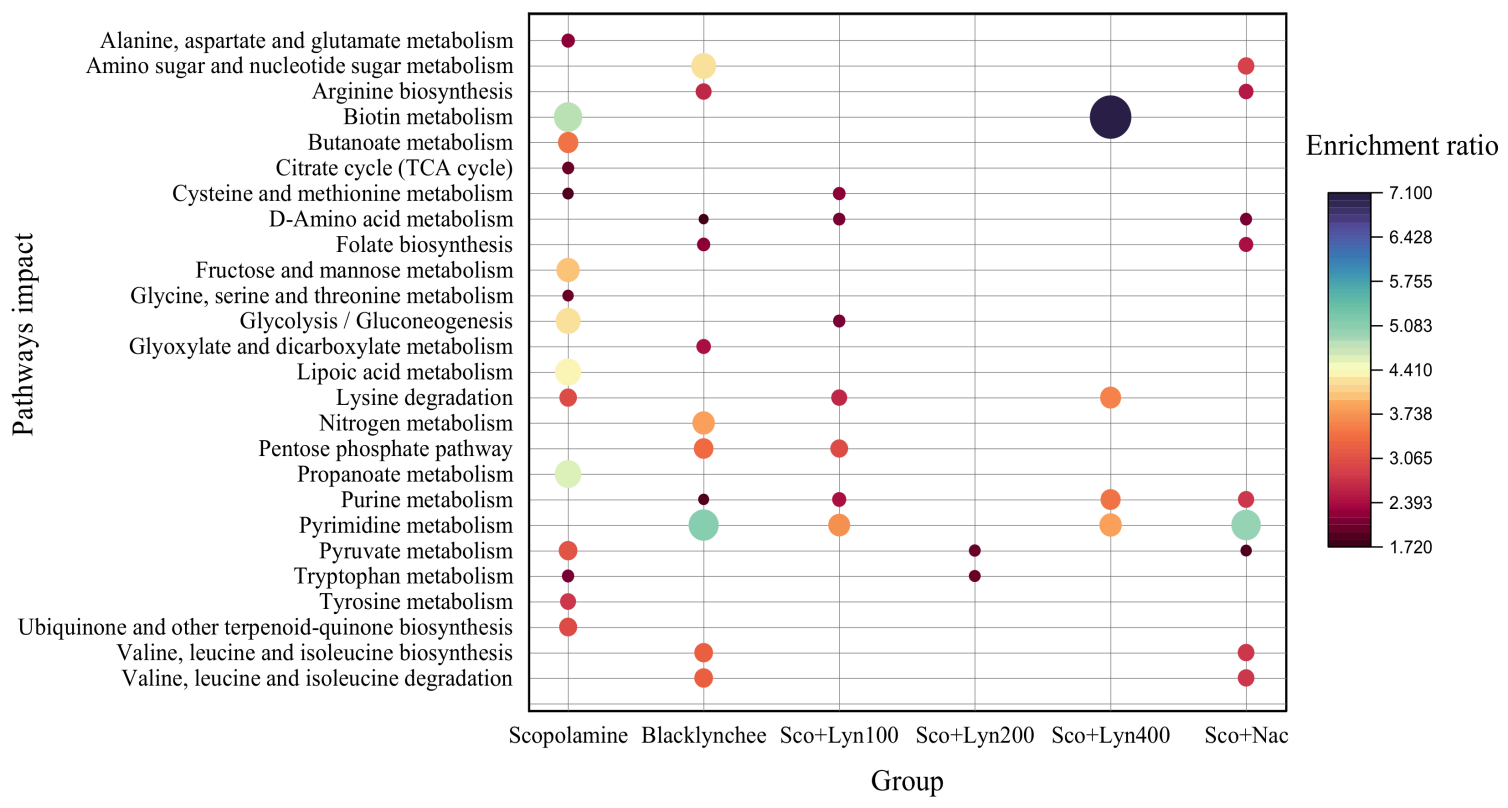
The bubble plot shows the metabolic pathways significantly altered in each experimental group compared with the control group. Bubble size represents pathway impact, while bubble color indicates pathway significance based on enrichment analysis. Larger and darker bubbles indicate pathways with greater biological impact and higher statistical significance.

Pathway enrichment analysis was performed on the significantly altered metabolites identified by ^1^H-NMR spectroscopy to investigate the biological processes affected by scopolamine and black lychee treatment (Figure 6). Compared with the control group, scopolamine administration markedly disrupted 26 metabolic pathways, indicating extensive metabolic disturbances associated with cognitive impairment. These altered pathways were mainly involved in oxidative stress, energy metabolism, amino acid metabolism, and neurotransmitter biosynthesis, demonstrating that scopolamine induced systemic metabolic dysfunction. Treatment with black lychee attenuated the scopolamine-induced metabolic disturbances. A comparable trend was observed in the NAC-treated group, supporting the neuroprotective effects of antioxidant intervention. Among the affected pathways, alanine, aspartate and glutamate metabolism, glycine, serine and threonine metabolism, arginine biosynthesis, pyruvate metabolism, and the TCA cycle exhibited the highest pathway impact values.

### 3.6. Correlation Between Blood Metabolites and Cognitive Performance

There were 15 blood metabolites significantly associated with the passive avoidance retention test (Table 2). Among these, 11 metabolites showed negative correlations, and 4 showed positive correlations with memory retention scores. Notably, N^6^-methyladenosine, quinolinic acid, and pentosidine exhibited moderate negative correlations with memory retention and have previously been implicated in cognitive dysfunction [26].

## 4. Discussion

This study shows that black lychee protects against cognitive decline in a scopolamine-induced rat model of cognitive impairment. In the passive avoidance test, rats treated with 100, 200, and 400 mg/kg black lychee showed longer step-through latency than the scopolamine group, indicating better memory retention. This behavioral improvement was linked to changes in the blood metabolic profile, which shifted closer to that of the control group. Specifically, black lychee partially reversed scopolamine-induced disturbances in oxidative stress, the kynurenine pathway, and energy metabolism. Together, these results suggest that black lychee improves memory through multiple metabolic mechanisms, supporting its potential as a functional food for preventing cognitive decline.

### 4.1. Phytochemical Profile of Black Lychee and Relevance to Cognitive Impairment

^1^H-NMR spectroscopic analysis identified seventeen phytochemicals in black lychee, consisting of flavan-3-ols, flavonols, anthocyanins, and phenolic acids. In blood, eight phytochemicals were identified, including catechin, epicatechin, epigallocatechin gallate, flavone, gallic acid, caffeic acid, quercetin, and kaempferol. The concentrations of gallic acid, caffeic acid, and flavone were significantly increased in the black lychee-treated groups compared with the control, SCO-only, and SCO + NAC400 groups.

This rich polyphenolic profile is consistent with previous reports on lychee and its thermal processing products. Such processing is known to promote the accumulation of low-molecular-weight phenolics through controlled Maillard and browning reactions [16]. Among the compounds detected in blood, gallic acid has been shown to inhibit amyloid-β aggregation and reduce oxidative stress via activation of the Nrf2/HO-1 pathway [27], while caffeic acid exerts complementary neuroprotective effects, including inhibition of acetylcholinesterase and butyrylcholinesterase, suppression of amyloid-β aggregation, and activation of the same Nrf2/HO-1 antioxidant pathway [28]. Catechin and epicatechin have been reported to protect neurons by scavenging reactive oxygen species and modulating neuroinflammatory signaling [29], whereas quercetin and its glycoside derivatives exert anti-neuroinflammatory effects by suppressing NF-κB activation and reducing pro-inflammatory cytokine production [30]. Notably, chlorogenic acid was identified in the black lychee but was not detected in blood; it has nonetheless been reported, together with caffeic acid and p-coumaric acid, to improve mitochondrial function and attenuate oxidative damage in neuronal cells [28,31,32]. The co-occurrence of these bioactive compounds, several of which converge on shared pathways such as Nrf2/HO-1 signaling and amyloid-β suppression, points to a complementary, multi-target mechanism of action that may be relevant to the multi-factorial pathophysiology of cognitive impairment [33].

Therefore, the beneficial effects observed in this study should be interpreted as the combined actions of multiple phytochemicals rather than the effect of a single major compound. Although gallic acid and caffeic acid were the predominant phenolic compounds detected in the blood, their individual contributions could not be distinguished in the present study.

### 4.2. Effect of Black Lychee on Locomotor Activity and Cognitive Performance

No significant differences in locomotor activity were observed among the experimental groups in the open-field test, confirming that the behavioral effects observed in the passive avoidance test were not attributable to motor impairment. This is consistent with previous reports that scopolamine at the dose used in this study selectively impairs central cholinergic function [34,35].

In the passive avoidance test, scopolamine administration significantly reduced step-through latency in the 24 h retention test compared with the control group, confirming the successful induction of memory impairment. Scopolamine acts as a competitive antagonist at muscarinic acetylcholine receptors, thereby disrupting cholinergic neurotransmission in the hippocampus and cortex, regions critical for learning and memory consolidation [36]. Black lychee treatment significantly improved retention latency at all doses tested, although the magnitude of improvement varied. The SCO + BL100 and SCO + BL400 groups both reached the maximum cut-off time of 300 s, whereas the SCO + BL200 group showed a smaller, though still significant, improvement (247.4 ± 98.2 s). These results indicate that black lychee effectively attenuated scopolamine-induced cognitive impairment across the tested dose range. The positive control group treated with NAC similarly showed improved latency (265.6 ± 85.3 s), supporting the role of antioxidant mechanisms in cognitive protection [37].

The observed negative correlation between memory performance and circulating levels of quinolinic acid, pentosidine, and $N^{6}$-methyladenosine is biologically plausible. Scopolamine-induced cholinergic blockade is known to trigger systemic oxidative stress, neuroinflammation, and metabolic disruption. Elevated quinolinic acid reflects activation of the neurotoxic kynurenine pathway [38], whereas increased pentosidine serves as a biomarker of oxidative stress-induced advanced glycation end product (AGE) formation [39]. Additionally, alterations in $N^{6}$-methyladenosine indicate cellular stress responses at the epitranscriptomic level [40]. The reduction in these systemic metabolites by black lychee supports its role in mitigating scopolamine-induced metabolic and oxidative stress.

### 4.3. Blood Metabolomic Alterations Induced by Scopolamine and Modulation by Black Lychee

Metabolomic profiling revealed that scopolamine induced widespread disruption across multiple essential biochemical pathways and that treatment with black lychee attenuated these disturbances, shifting the metabolic profile toward that of the control group. PCA and PLS-DA of blood metabolomic data revealed distinct separation between the scopolamine group and the control group, with a significant overall treatment effect confirmed by PERMANOVA (F = 2.67, *p* = 0.001, R^2^ = 0.25). Although the PLS-DA model showed acceptable goodness-of-fit (R^2^ = 0.7149), its predictive ability was moderate (Q^2^ = 0.3186), and permutation testing indicated the possibility of model overfitting. Therefore, the multivariate discrimination should be interpreted with caution. To reduce reliance on a single statistical approach, the biological interpretation was supported by complementary analyses, including univariate statistical testing, volcano plot analysis, and pathway enrichment.

Although 25% of the total variance was explained by the treatment conditions, this level of explained variance is consistent with the inherent biological variability expected in systemic blood metabolomics studies and reflects the complex, multi-pathway nature of scopolamine-induced metabolic disruption. VIP analysis identified L-lactic acid as the most discriminative metabolite (VIP score > 2.8). Creatinine, α-ketoglutarate, glycerol-3-phosphate, and ATP were markedly reduced in the scopolamine group compared with controls, while L-lactic acid levels were elevated, suggesting a shift toward anaerobic energy metabolism and increased oxidative stress following cholinergic disruption [41]. Black lychee treatment improved systemic metabolic homeostasis by attenuating scopolamine-induced metabolic disturbances. Volcano plot analysis further confirmed that the number of significantly dysregulated metabolites was reduced at all doses of black lychee. The metabolic restoration observed in the NAC-treated group was comparable to that of the black lychee groups, supporting the hypothesis that antioxidant activity is a key mechanism by which black lychee exerts its neuroprotective effects.

### 4.4. Metabolic Pathway Analysis

Pathway enrichment analysis revealed that scopolamine administration significantly induced oxidative stress and altered the kynurenine pathway and energy metabolism, particularly pyruvate metabolism and the TCA cycle. Scopolamine administration induced lipid peroxidation and depleted reduced glutathione, whereas treatment with black lychee effectively reduced oxidative stress. Concurrently, scopolamine-induced neuroinflammation depleted both tryptophan and picolinic acid, diverting tryptophan metabolism toward the neurotoxic kynurenine pathway.

Intervention with black lychee produced distinct, dose-dependent metabolic shifts. The 100 mg/kg dose elevated both tryptophan and picolinic acid levels. The 200 mg/kg dose increased only picolinic acid, but the 400 mg/kg dose caused a significant drop in picolinic acid without altering tryptophan levels. This non-monotonic dose–response pattern warrants further investigation, particularly at higher doses.

Disruption of the TCA cycle and pyruvate metabolism reflects impaired mitochondrial energy production. When mitochondrial function is impaired, brain cells can no longer efficiently generate ATP through oxidative phosphorylation and instead rely more heavily on anaerobic glycolysis, consistent with the elevated L-lactic acid levels observed in the scopolamine group. Lactic acid is produced primarily by astrocytes in the brain, where it serves as an energy substrate shuttled to neurons [42]. Both a deficiency and an excessive accumulation of lactic acid can disrupt normal neurological function, and this dysregulation has been linked to the development of cognitive impairment [41]. Notably, black lychee administration reversed these metabolic deficits in scopolamine-treated rats by lowering lactic acid levels, elevating ATP production, and restoring key metabolites involved in energy metabolism.

Black lychee is rich in phenolic compounds and other bioactive constituents that possess antioxidant, anti-inflammatory, and neuroprotective properties. Phytochemicals are thought to attenuate oxidative stress [43] and preserve neuronal function, thereby contributing to the improvement of cognitive impairment. However, the thermal processing used to produce black lychee inevitably promotes the Maillard reaction, generating undesirable by-products, especially AGEs [44]. These by-products may be associated with oxidative stress, inflammation, and metabolic dysfunction [45]. Consequently, a higher intake of black lychee may not proportionally enhance its neuroprotective efficacy, despite the potential benefits conferred by its phenolic compounds.

In the present study, the 100 mg/kg/day dose produced favorable improvements in memory performance and metabolic alterations. These findings suggest that lower-dose supplementation may provide an appropriate balance between efficacy and minimizing exposure to potentially harmful processing-derived by-products. Future studies should elucidate the mechanisms of black lychee action in brain tissue, including the bioavailability of phenolic compounds and processing-derived constituents, in order to confirm its beneficial effects and optimize the development of black lychee as a functional food for cognitive protection.

### 4.5. Limitation

Several limitations should be considered when interpreting the findings of this study. First, the scopolamine-induced model is widely used for cognitive impairment investigation and cholinergic dysfunction. However, it represents an acute pharmacological model and does not fully recapitulate the complex pathological features of Alzheimer’s disease, including amyloid deposition, tau pathology, progressive neurodegeneration, and synaptic loss. Therefore, the metabolic alterations observed in this study should be interpreted as responses associated with scopolamine-induced cognitive impairment rather than direct evidence of Alzheimer’s disease pathology. Second, the present study focused only on blood metabolomic profiling, and brain metabolomic alterations were not directly investigated. Therefore, the relationship between peripheral metabolic changes and central nervous system alterations remains unclear. Future studies incorporating brain metabolomics, neurotransmitter quantification, histopathological evaluation, and molecular analyses will be necessary to further validate the proposed mechanisms underlying the neuroprotective effects of black lychee supplementation. Third, although behavioral improvements were observed, the absence of molecular and biochemical validation, including assessment of acetylcholinesterase activity, oxidative stress markers, inflammatory mediators, neurotrophic factors, synaptic proteins, and hippocampal pathology, limits the ability to establish specific biological mechanisms. These complementary analyses should be incorporated into future investigations to strengthen mechanistic interpretation. Finally, multiple statistical analyses were performed in this study, including metabolite screening, volcano plot analysis, VIP analysis, pathway enrichment analysis, and correlation analysis. False discovery rate (FDR) correction was not applied across all analyses, and therefore, some findings should be considered exploratory. The identified metabolites and metabolic pathways should be further validated in independent cohorts and using complementary analytical approaches.

## 5. Conclusions

This study demonstrated that black lychee, which contains polyphenolic compounds, exerted neuroprotective effects in a scopolamine-induced rat model of cognitive impairment. Untargeted ^1^H-NMR metabolomics revealed that black lychee supplementation improved cognitive performance and reduced systemic metabolic disturbances. The 100 mg/kg/day dose may confer greater neuroprotective and metabolic benefits than higher doses. These findings support the potential of black lychee as a functional food for mitigating scopolamine-induced cognitive impairment. The elevated blood levels of gallic acid and caffeic acid observed following black lychee supplementation, combined with their previously reported neuroprotective mechanisms, provide a plausible mechanistic basis for the cognitive and metabolic benefits observed in this study. These results also provide a rationale for further optimization of processing conditions to maximize beneficial phytochemicals while minimizing undesirable processing-derived compounds.

## Figures and Tables

**Table 1:** Effects of black lychee on locomotor activity and passive avoidance performance in scopolamine-induced rats.

Groups	Number of Line Crossings	Trial 1 Training Session (S)	Trial 2 Retention Test After 24 h (S)
Control	47.4 ± 18.7	24.7 ± 8.6	300
Scopolamine	41.5 ± 17.9	17.4 ± 6.6	142.3 ± 54.9 *
BL 400	36.6 ± 19.1	29.9 ± 10.6	241.5 ± 78.6
SCO + BL100	50.8 ± 9.5	62.9 ± 23.7	300
SCO + BL200	55.8 ± 11.4	69.1 ± 24.4	247.4 ± 98.2
SCO + BL400	64.0 ± 9.0	39.8 ± 16.4	300
SCO + NAC	48.6 ± 16.8	46.3 ± 6.4	265.6 ± 85.3

Data are presented as mean ± SD (n = 8 per group). Locomotor activity was assessed by counting the number of line crossings during a 300 s open-field test. Passive avoidance performance (cut-off time: 300 s) was evaluated based on step-through latency during the training session (Trial 1) and the retention test 24 h post-acquisition (Trial 2), where a longer latency indicates superior learning and memory retention. Statistical analysis was performed using the Kruskal–Wallis test followed by Dunn’s post hoc test. * *p* < 0.05 compared with the other groups.

**Table 2:** Spearman’s rank correlation coefficients between significantly correlated blood metabolites and step-through latency in the passive avoidance retention test.

Metabolites	Spearman Correlation (ρ)	*p*-Value	Metabolites	Spearman Correlation (ρ)	*p*-Value
N^6^-methyladenosine	−0.281	0.037	L-acetylcarnitine	−0.323	0.016
L-cystathione	−0.305	0.023	L-glutamic acid	−0.267	0.049
Phosphorylcholine	−0.317	0.018	Lanthionine	−0.335	0.012
L-alanine	0.419	0.001	2-phosphoglyceric acid	0.311	0.021
Citrulline	0.278	0.04	6-phosphoglucunic acid	−0.304	0.024
Indoxyl	−0.31	0.021	Myo-inositol	0.286	0.034
Quinolinic acid	−0.335	0.013	Pentosidine	−0.308	0.022
N-acetyl-aspartate-glutamate	−0.267	0.049			

## Data Availability

The datasets generated and/or analyzed during the current study are available from the corresponding author on reasonable request.

## Supplementary Materials

The following supporting information can be downloaded at: https://www.mdpi.com/article/10.3390/biology15151325/s1, Table S1: Fold changes of blood metabolites associated with oxidative stress, the kynurenine pathway, and energy metabolism in each treatment group relative to the control group; Table S2: Fold changes of blood metabolites associated with oxidative stress, the kynurenine pathway, and energy metabolism in each treatment group relative to the scopolamine group; Table S3: Relative abundances of blood phytochemicals in each treatment group determined by 1H-NMR spectroscopy; Figure S1. Partial least squares-discriminant analysis (PLS-DA) of serum metabolomic profiles from the seven experimental groups. (A) PLS-DA score plot showing the distribution of samples according to treatment groups. (B) Internal cross-validation of the PLS-DA model (R^2^ = 0.7149, Q^2^ = 0.3186). (C) Permutation test (200 permutations) used to assess the robustness of the PLS-DA model.

## Abbreviations

The following abbreviations are used in this manuscript:

^1^H-NMR	Proton nuclear magnetic resonance spectroscopy
AGE	Advanced glycation end products
BL	Black lychee
C	The metabolite concentration
CPMG	Carr–Purcell–Meiboom–Gill
GBD	Global Burden of Disease
H	The number of protons
HMDB	Human Metabolome Database
I	Integrated peak intensity
NAC	N-acetyl-L-cysteine
PCA	Principal component analysis
PLS-DA	Partial least squares-discriminant analysis
SCO	Scopolamine
TCA	Tricarboxylic acid cycle
TSP	3-(Trimethylsilyl) propionic-2,2,3,3-d_4_ acid sodium salt
VIP	Variable importance in projection
