# Supplementary material for: Blood Metabolomic Profiling of Systemic Responses to Dried Black Lychee in a Scopolamine-Induced Cognitive Impairment in Rats
Source: Biology (Basel). 2026 Aug 6;15(15):1325. doi: 10.3390/biology15151325 (PMC13465933; doi:10.3390/biology15151325)
Supplement: Supplementary file 1 [file biology-15-01325-s001.zip › biology-4437522-supplementary.pdf]

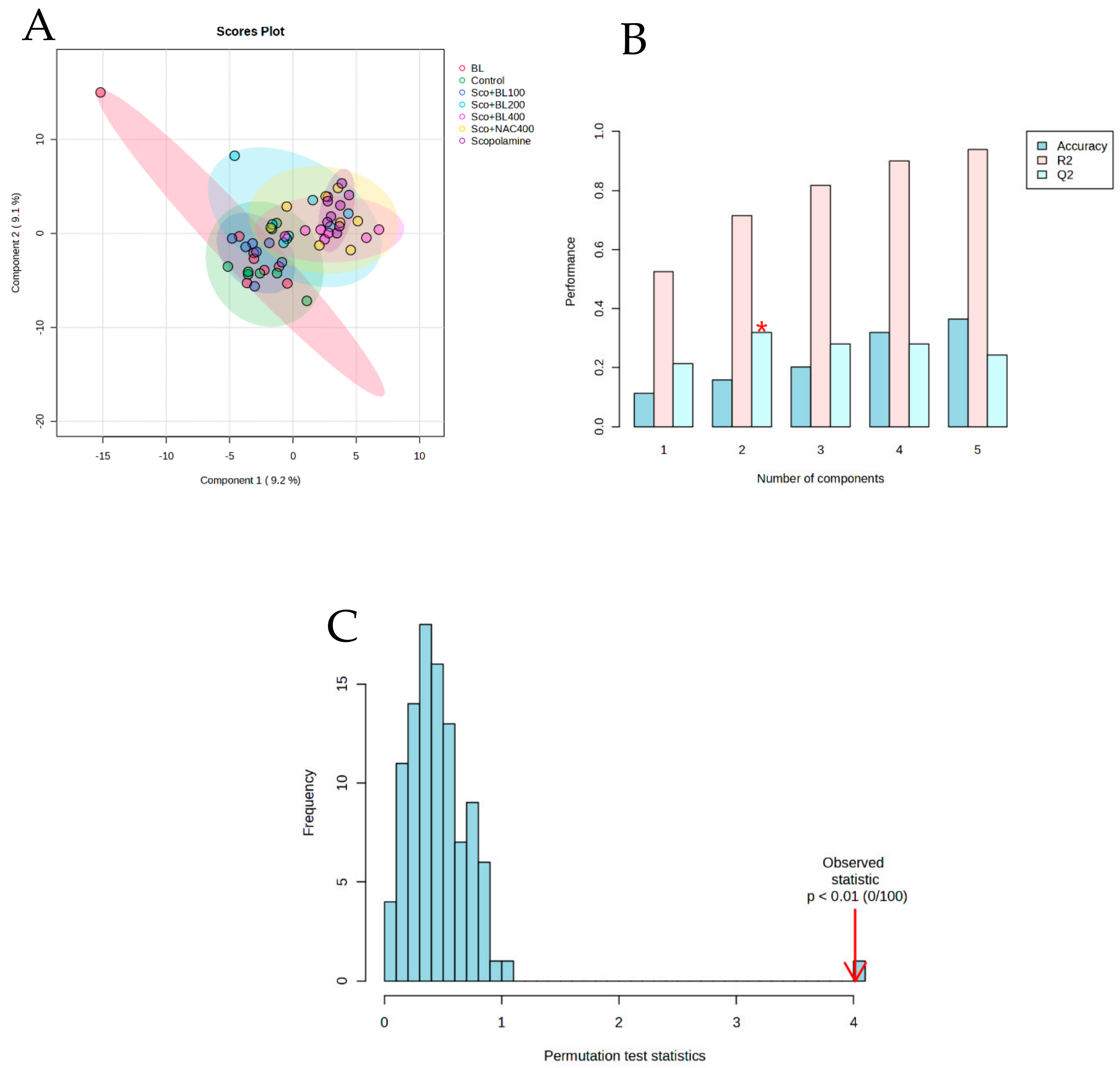

Figure S1. Partial least squares-discriminant analysis (PLS-DA) of serum metabolomic profiles from the seven experimental groups. (A) PLS-DA score plot showing the distribution of samples according to treatment groups. (B) Internal cross-validation of the PLS-DA model ($R^2 = 0.7149$, $Q^2 = 0.3186$). (C) Permutation test (200 permutations) used to assess the robustness of the PLS-DA model.

Table S1. Fold changes of blood metabolites associated with oxidative stress, the kynurenine pathway, and energy metabolism in each treatment group relative to the control group.

Metaboloites	SCO	BL400	SCO+BL100	SCO+BL200	SCO+BL400	SCO+NAC400
8-OHdG	1.01	0.96	1.12	0.98	1.24	1.08
Pentosidine	0.86	1.00	1.36	0.76	0.81	1.12
Malondialdehyde	1.20	1.47	1.36	1.25	1.20	1.34
Sarcosine	0.92	0.98	0.98	0.94	1.08	1.13
Anserine	1.08	1.28	1.08	1.09	0.93	0.92
Oxidized-glutathione	0.96	1.24	0.92	0.91	1.02	0.99
Glutathione	0.91	0.88	0.87	1.00	0.88	0.94
L-glutamic acid	1.23	1.22	1.07	0.96	0.99	1.03
L-glutamine	1.17	1.37	1.38	1.09	1.42	1.03
L-Cysteine	1.13	0.98	0.92	1.03	1.03	1.06
Cystathione	0.86	1.25	1.26	0.88	1.05	0.74
L-Cystine	1.12	0.86	0.94	1.03	1.27	1.11
N-acetyl-aspartyl-glutathione	0.86	0.94	0.96	0.98	0.98	1.02
Quinolinic acid	0.64	0.74	0.88	0.88	0.63	0.47
Kynulinic acid	1.29	1.09	1.33	1.06	1.13	1.32
Kynurenine	0.79	1.00	0.98	0.86	0.96	0.90
Picolinic acid	0.78	1.18	1.25	1.04	0.48	1.10
Anthranillic acid	1.24	1.51	1.63	1.11	1.17	1.48
3-Hydroxykynurenine	0.87	0.93	0.95	0.92	0.82	0.91
Tryptophan	0.85	0.84	0.89	0.82	0.95	0.90
Malic acid	0.96	1.07	1.05	0.83	0.89	1.09
Pyruvate	1.30	1.02	1.00	0.95	1.05	1.18
Pyroglutamic acid	0.87	0.75	0.66	1.20	0.81	0.72
Succinic acid	1.34	1.12	0.87	1.21	0.96	0.97
Isocitric acid	1.13	1.10	1.17	0.82	1.09	1.17
Citric acid	1.47	1.68	1.25	0.98	1.00	1.20
Creatinine	0.76	0.95	1.13	0.79	0.82	0.79
A-Ketaglutarate	0.70	0.95	0.88	0.99	0.79	0.50
Ornitine	1.10	1.14	1.16	0.83	1.21	1.22
Acetyl- Co A	0.91	1.11	1.40	1.21	0.589	1.21
L-carnitine	1.06	1.00	0.96	0.98	0.98	0.97
Oxaloacetic acid	1.00	1.03	0.85	0.84	0.95	0.84
Glycero-3-phosphate	0.85	1.12	0.97	0.87	1.22	1.13
Creatinine	1.14	1.37	1.45	0.77	1.32	1.48
Lactic acid	30.92	0.92	0.73	7.73	2.27	1.99
ATP	0.81	0.99	1.04	0.91	0.94	0.75
Fumaric acid	0.90	0.81	0.91	0.66	0.81	0.57

Fold changes were calculated by dividing the mean metabolite level in each treatment group by that of the control group. Values >1 indicate increased metabolite levels, whereas values <1 indicate decreased metabolite levels relative to the control group.

Table S2. Fold changes of blood metabolites associated with oxidative stress, the kynurenine pathway, and energy metabolism in each treatment group relative to the scopolamine group.

Metaboloites	BL400	SCO+BL100	SCO+BL200	SCO+BL400	SCO+NAC400
8-OHdG	0.95	1.11	0.97	1.22	1.07
Pentosidine	1.15	1.57	0.88	0.94	1.29
Malondialdehyde	1.22	1.13	1.04	1.00	1.20
Sarcosine	1.07	1.07	1.03	1.17	1.23
Anserine	1.18	1.00	1.01	0.86	0.85
Oxidized-glutathione	1.31	0.95	0.94	1.05	1.02
Glutathione	0.96	0.95	1.09	0.96	1.03
L-glutamic acid	0.98	0.87	0.76	0.81	0.83
L-glutamine	1.16	1.18	0.92	1.21	1.23
L-Cysteine	0.86	0.83	0.91	0.92	0.91
Cystathione	0.80	1.53	1.02	0.95	0.86
L-Cystine	0.76	0.83	0.92	1.14	0.99
N-acetyl-aspartyl-glutathione	1.09	1.12	1.14	1.14	1.18
Quinolinic acid	1.15	1.37	1.37	0.98	0.73
Kynulinic acid	0.84	1.03	0.82	0.88	1.02
Kynurenine	1.28	1.25	1.11	1.24	1.15
Picolinic acid	1.50	1.59	1.32	0.49	1.41
Anthranillic acid	1.21	1.32	0.90	0.95	1.19
3-Hydroxykynurenine	1.00	1.05	1.10	1.00	1.09
Tryptophan	0.99	1.37	0.97	1.12	1.06
Malic acid	1.11	1.09	0.86	0.92	1.12
Pyruvate	0.78	0.77	0.73	0.81	0.76
Pyroglutamic acid	0.86	0.76	1.38	0.93	0.83
Succinic acid	0.84	0.65	0.91	0.72	0.72
Isocitric acid	0.96	1.02	0.72	0.95	1.02
Citric acid	1.14	0.85	0.67	0.68	0.81
Creatinine	1.23	1.49	1.03	1.07	1.03
A-Ketaglutarate	1.36	1.26	1.41	1.12	0.71
Ornitine	1.04	1.06	0.76	1.10	1.11
Acetyl- Co A	1.21	1.52	1.31	0.96	1.31
L-carnitine	0.95	0.90	0.91	0.92	1.29
Oxaloacetic acid	1.02	0.84	0.83	0.94	0.84
Glycero-3-phosphate	1.31	1.14	1.01	1.43	1.32
Creatinine	1.20	1.27	0.68	1.16	1.29
Lactic acid	0.02	0.02	0.24	0.07	0.06
ATP	1.22	1.28	1.12	1.15	0.91
Fumaric acid	0.91	1.01	0.73	0.90	0.63

Fold changes were calculated by dividing the mean metabolite level in each treatment group by that of the scopolamine group. Values >1 indicate increased metabolite levels, whereas values <1 indicate decreased metabolite levels relative to the scopolamine groups.

Table S3. Relative abundances of blood phytochemicals in each treatment group determined by ^{1}H-NMR spectroscopy.

Phytochemicals (µM)	Control	SCO	BL400	SCO+BL100	SCO+BL200	SCO+BL400	SCO+NAC
Catechin	2.30±0.32	2.43±0.26	2.53±0.14	2.92±0.25	2.76±0.42	2.75±0.33	3.03±0.33
epicatechin	1.29±0.38	1.36±0.34	1.37±0.41	1.69±0.33	1.59±0.33	1.48±0.18	1.64±0.15
epigcllocatechin gallate	0.95±0.20	1.05±0.22	1.05±0.18	1.16±0.11	1.12±0.19	1.10±0.13	1.13±0.11
Flavone	1.95±0.95	2.61±2.18	2.71±2.44	3.55±3.58	9.41±3.66*	9.00±4.73*	3.72±3.88
gallic acid	3.93±1.01	3.37±0.80	6.47±1.07*	6.47±0.96*	6.26±1.01*	6.94±1.32*	3.95±0.63
caffeic acid	2.67±2.67	2.67±0.74	5.34±0.42*	5.64±0.68*	5.13±1.11*	5.60±0.77*	2.80±0.38
quercetin	1.07±0.34	1.46±0.32	1.51±0.25	1.76±0.38	1.73±0.36	1.82±0.29	1.97±0.27
kaempferol	0.50±0.27	0.98±0.18	0.88±0.11	1.07±0.24	0.99±0.17	0.96±0.18	1.04±0.28

Data are presented as median (interquartile range, IQR) (n = 8 per group). Statistical analysis was performed using the Kruskal–Wallis test followed by Dunn's multiple comparisons test. Values with different superscript letters indicate significant differences among groups ($p < 0.05$).
